# Facing the COVID-19 Pandemic: A Mixed-Method Analysis of Asylum Seekers’ Experiences and Worries in the Canton of Vaud, Switzerland

**DOI:** 10.3389/ijph.2023.1606229

**Published:** 2023-09-27

**Authors:** Kevin Morisod, Tiffany Martin, Cloé Rawlinson, Véronique S. Grazioli, Christian von Plessen, Marie-Anne Durand, Kevin Selby, Marie-Annick Le Pogam, Nolwenn Bühler, Patrick Bodenmann

**Affiliations:** ^1^ Department of Vulnerabilities and Social Medicine, University Center of General Medicine and Public Health, Lausanne, Switzerland; ^2^ Chair of Medicine for Vulnerable Populations, Faculty of Biology and Medicine, University of Lausanne, Lausanne, Switzerland; ^3^ Department of Epidemiology and Health Systems, Center for Primary Care and Public Health (Unisanté), Lausanne, Switzerland; ^4^ Department of Ambulatory Care, Center for Primary Care and Public Health, University Center of General Medicine and Public Health, Lausanne, Switzerland; ^5^ Direction Générale de la Santé (DGS), Lausanne, Switzerland; ^6^ Department of Clinical Research, University of Southern Denmark, Odense, Denmark; ^7^ UMR1295 Centre d’Epidémiologie et de Recherche en Santé des Populations (CERPOP), Toulouse, France; ^8^ University Center of General Medicine and Public Health, Lausanne, Switzerland; ^9^ The Dartmouth Institute for Health Policy and Clinical Practice, Dartmouth College, Lebanon, NH, United States; ^10^ Institute of Social Sciences, Faculty of Social and Political Sciences, University of Lausanne, Lausanne, Switzerland

**Keywords:** COVID-19, experiences, worries, mixed-methods study, asylum seekers, community health

## Abstract

**Objectives:** The clinical and social burden of the COVID-19 pandemic were high among asylum seekers (ASs). We aimed to understand better ASs’ experiences of the pandemic and their sources of worries.

**Methods:** Participants (*n* = 203) completed a survey about their worries, sleep disorders, and fear of dying. We also conducted semi-structured interviews with ASs living in a community center (*n* = 15), focusing on how social and living conditions affected their experiences and worries.

**Results:** ASs in community centers experienced more sleep disorders related to the COVID-19 pandemic than those living in private apartments (aOR 2.01, *p* = 0.045). Similarly, those with lower education had greater fear for their life due to the COVID-19 pandemic (aOR 2.31, *p* = 0.015). Qualitative findings showed that sharing living spaces was an important source of worries for ASs and that protective measures were perceived to increase social isolation.

**Conclusion:** Our study highlighted the impact of the COVID-19 pandemic for ASs and the importance of tailoring public health measures to their needs and living conditions.

## Introduction

The clinical and social burden of the COVID-19 pandemic was notably high among migrant populations–especially asylum seekers and refugees worldwide [1, 2]. A systematic review found, for example, that asylum seekers and refugees were at increased risk of infection, hospitalization, and higher mortality [3]. High population density, belonging to a minority ethnic group, or social deprivation were all identified as risk factors for contracting SARS-CoV-2 infection [4–7]. Moreover, the impact of inappropriate COVID-19 public health measures disproportionally affected migrant populations [8–11]. For example, in a large international survey, authors found that refugees who had more difficulties accessing COVID-19 preventive measures had worse mental health and faced more discrimination [12].

These effects were stronger for those with more insecure housing and residence status, highlighting the need to consider social context and living conditions in the management of the COVID-19 pandemic [13]. Yet, migrant populations are not a homogenous group. Some communities seem to have been particularly exposed during this pandemic due to poor social determinants of health, such as undocumented migrants and those living in community centers [14–18]. Community centers are, indeed, characterized by a high population density, shared rooms, and little or no privacy, factors that may have amplified the negative experience of the pandemic for the resident populations and increased their worries [19].

Similarly, asylum seekers with limited awareness or a lack of understanding of public health recommendations due to inadequate communication and language or cultural barriers may have experienced more worries about the COVID-19 pandemic (fear of being infected or dying) and greater mental health deterioration [20, 21]. Previous literature has indeed identified inequities surrounding communication during pandemics, affecting linguistic minorities and socially excluded. This unequal access to information created mistrust, causing stress, anxiety and apprehension in the face of a pandemic [22].

Therefore, our goal was to measure and understand asylum seekers’ pandemic experiences and worries. Specifically, we aimed to explore and deepen the understanding of ASs’ experiences of the COVID-19 pandemic according to their living conditions and other social factors, such as immigrant status, education level, language proficiency and health literacy.

## Methods

### Design

We applied a sequential explanatory mixed method design, i.e., we started with a quantitative survey followed by qualitative semi-directed interviews to explain the survey results [23]. Interview participants were not among those who completed the survey.

### Participants

According to cantonal administrative data, 744 asylum seekers (ASs) were living in one of the ten community centers of the Canton of Vaud in October 2020, i.e., ten percent of ASs in Switzerland.

Study participants were ASs with a pending procedure, temporarily admitted (with a residence permit), or rejected (without a residence permit). ASs with a permit have access to the labor market. We excluded children, individuals not living in the Canton of Vaud, and former refugees who had obtained a settlement permit. We also excluded ASs unable to read or write.

### Procedures

#### Recruitment

We distributed the questionnaire in ten ASs’ centers from the Canton of Vaud between August and October 2020. We conducted qualitative interviews in one of them from spring to the end of summer 2021. For both quantitative and qualitative data collection, participants were contacted by the center staff. This approach facilitated access to this population, usually under-represented in research and particularly difficult to reach during the pandemic.

During data collection, face masks were mandatory within community centers, protective measures (i.e., hand hygiene, social distancing, and limited contacts) were recommended, and positive cases for SARS-CoV-2 were subject to quarantine and isolation. In the Canton of Vaud, vaccination was available and free of charge for people over 18 years, including ASs, from April 2021.

The center where the interviews were conducted is located between the industrial area and a forest on the periphery of the major city of the Canton. It is geographically and socially isolated, with no residential areas nearby. The center has several buildings and can accommodate up to 296 people when running at maximal capacity. During data collection, 250 people were living in the center. Most rooms were shared and measured twelve square meters (single rooms were less than 9 square meters). Facilities included one bathroom and one kitchen for about 18 residents.

#### Quantitative Assessment (Surveys)

Participants completed a self-administrated cross-sectional survey about their experiences and worries with the COVID-19 pandemic. The (online and paper) questionnaires were developed using REDCap (Supplementary Material S1).

#### Qualitative Assessment (Interviews)

We conducted thirteen semi-structured face-to-face interviews with 15 participants at one of the community centers, including five women and ten men (Supplementary Material S2). These were individual or group interviews (i.e., one with the participant’s partner and one with a participant’s friend). Most were conducted in French, two in English, one in Spanish, and four in the participant’s language of origin with the help of a professional interpreter. The interviews lasted between 27 and 90 min (mean 58 min, SD 17.2 min). They were recorded on a smartphone and transcribed literally.

In addition, we interviewed the center manager, a nurse, and a social worker to understand how they dealt with the COVID crisis and the challenges they faced, to gain a different perspective on the experiences of ASs. For this article, we have only analyzed ASs’ interviews because we wanted to account for their subjective experiences of worries.

### Quantitative Measures

The questionnaire was adapted from a previously used online survey conducted on the general population of the Canton of Vaud [24]. First, with the help of a group of experts, we adapted the questions for a lower English reading level. Thus, bilingual medical and nursing students from a local nonprofit organization and community interpreters translated the questions into the nine most common languages among ASs residing in the Canton of Vaud: French, Tigrinya, Dari, Arab, Somali, Georgian, Tamil, Albanian, and Serbo-Croatian. Except for Tigrinya and Tamil, a second translator proofreads each translation.

#### Socio-Demographic Characteristics, Health Literacy, and COVID-19-Related Measures

A set of single items assessed socio-demographic characteristics, including age (in years), gender (male vs. female), level of education (low vs. high), French language proficiency (low vs. high), adapted and translated versions of a validated health literacy item (low vs. high) [25], type of residence (community centers vs. private apartments), legal status (with or without a resident permit) and contact with a social worker (yes vs. no). Then, participants had to answer whether they had tested positive for COVID-19, were in a medically at-risk group (i.e., people over 65 years with comorbidities such as hypertension, diabetes, heart or lung problems, or a weakened immune system), and knew what to do if they had COVID-19 symptoms.

#### Experiences and Worries During the COVID-19 Pandemic

In a multiple-choice question (9 items), we first asked participants to identify the main consequences of the COVID-19 pandemic protective measures on their daily lives. Then, participants were asked to indicate their degree of worry about the COVID-19 pandemic. They scored “general worry” and “worry about poor access to care” on a Likert-type scale (0–10, with 0 indicating “no worry at all” and 10 “extremely worried”). They also scored the magnitude of death fear and sleep disturbance associated with the COVID-19 pandemic using a 5-point Likert-type scale (Supplementary Material S1).

### Qualitative Measure

We conducted semi-directed interviews using an interview guide (Supplementary Material S3) to explore ASs’ experiences of the pandemic and its impact on different aspects of their daily life (including their migration process).

### Data Analysis

#### Quantitative Analysis (Surveys)

We described study participants’ characteristics and outcomes using frequencies (n) and relative frequencies (percentage) for dichotomous and categorical variables and median and interquartile ranges (IQR) for non-normally distributed continuous variables (mean and standard deviation otherwise).

We used logistic regressions to explore associations between the outcomes of interest and participant characteristics, such as place of living (community center or private apartment), legal status (ASs with a permit vs. rejected ASs), health literacy (high vs. low health literacy), education level (high vs. low education level) or official language proficiency (high vs. low French proficiency). Regression models were adjusted for age, gender and relevant confounders. We also conducted subgroup analysis by gender. Models’ calibration was tested using the Hosmer–Lemeshow and Pearson goodness-of-fit test. Associations with a *p*-value < 0.05 were considered statistically significant. Interactions between independent variables were assessed with the Chi-square test. Missing values were assumed to be missing at random. We compared with and without imputation of missing data, but no significant difference in the overall results was found. Hence, this paper is presented without imputation of missing data. All analyzes were performed with STATA version 16.

#### Qualitative Analysis (Interviews)

We performed inductive thematic analysis on the interview transcripts [26]. First, we reviewed the interviews to identify recurring categories, including ASs’ experiences and worries. Then, we used the qualitative data analysis software MAXQDA (release 22.1.1) to code the interview transcripts and perform systematic analysis. We reviewed the codes and discussed them during regular research team meetings.

## Results

### Quantitative Results (Surveys)

In total, 203 persons participated in the study. About two-thirds were men (*n* = 121), with a median age of 30 (IQR 23–39). More than half of the participants (58%, *n* = 118) lived in a community center, and 42% (*n* = 85) in a private apartment. Regarding legal status, 138 participants reported having a permit (70%), and 58 mentioned being without legal status (30%). Health literacy was low in 43% of the participants (*n* = 85), and 37% (*n* = 74) had a low level of education (compulsory or no education). In addition, 34% (*n* = 68) of participants described a low level of French comprehension (Table 1).

**TABLE 1:** Characteristics of survey participants (n = 203) Switzerland, October 2020.

Characteristics	Total, *n* (%)	Female, *n* (%)	Male, *n* (%)
Age (years)			
18–39	148 (75)	57 (74)	89 (75)
40–59	40 (20)	16 (21)	24 (20)
≥60	9 (5)	4 (5)	5 (4)
Gender			
Female	80 (40)	80 (100)	0 (0)
Male	121 (60)	0 (0)	121 (100)
Legal status			
Asylum seekers with permit	138 (70)	61 (80)	76 (64)
Asylum seekers without a permit	58 (30)	15 (20)	42 (36)
Education level			
High (University or high school)	78 (39)	29 (38)	49 (41)
Middle (Apprenticeship)	46 (23)	14 (18)	32 (27)
Low (Compulsory)	74 (37)	34 (44)	38 (32)
Health literacy^a^			
High	113 (57)	46 (60)	65 (55)
Low	85 (43)	31 (40)	54 (45)
Type of residence			
Community center	118 (58)	47 (59)	69 (57)
Private apartment	85 (42)	33 (41)	52 (43)
French language proficiency^b^			
High	132 (66)	50 (64)	81 (68)
Low	68 (34)	28 (36)	39 (32)
Tested for COVID-19			
Positive	5 (2.5)	2 (2.5)	3 (2.5)
Negative	23 (11.5)	6 (7.5)	17 (14)
Awaiting result	3 (1.5)	0	3 (2.5)
No	165 (82)	69 (87)	95 (78.5)
Don’t know	5 (2.5)	2 (2.5)	3 (2.5)
Social worker or community help			
Yes	80 (41)	25 (32)	54 (45)
No	117 (59)	52 (68)	65 (55)
At risk of medical complications (at least one comorbidity)			
Yes	31 (15)	13 (16)	17 (14)
No	170 (85)	67 (84)	103 (86)

^a^ Dichotomized, “Often” and “Always” as high and “Never,” “Rarely,” “Sometimes” and “I do not know” as low health literacy.

^b^ Dichotomized, “Very well” and “Well” as high, and “Not well,” “Not at all” and “I do not know” as low French language proficiency.

#### Impact of COVID-19 Pandemic Measures on Daily Life

The main reported impact of COVID-19 pandemic measures among ASs were social isolation (0.36, 95% CI [0.30–0.43]), increase in loneliness (0.35, 95% CI [0.29–0.42]), increase in anxiety (0.32, 95% CI [0.25–0.38]) and economic losses (0.17, 95% CI [0.12–0.23]) (Table 2). Moreover, in multivariable analyses, ASs with high French proficiency and those living in single apartments were statistically more impacted economically than those with low French proficiency (aOR 0.2, *p* = 0.009, 95% CI [0.036–0.73]) and those living in community center (aOR 0.4, *p* = 0.045, 95% CI [0.16–0.98]). In the subgroup analysis, for males, the presence of a social worker was associated with lower social isolation (OR 0.41, *p* = 0.02, 95% CI [0.19–0.88]) and loneliness [OR 0.34, *p* = 0.01, CI [0.15–0.77]), but not for females. Females at risk of COVID-19 complications had more anxiety than those non at risk (OR 3.50, *p* = 0.046, CI [1.02–12.00]) (Supplementary Table S1).

**TABLE 2:** Non-adjusted Odd Ratio of participants’ characteristics and COVID-19 pandemic impact on social isolation, loneliness, anxiety and economic losses. (with 95% CI and *p*-value)^a^ Switzerland, October 2020.

	Social isolation^b^	Loneliness	Anxiety	Economic losses
Gender (female)	0.69 (0.38–1.26, *p* = 0.23)	1.01 (0.56–1.83, *p* = 0.97)	1.39 (0.77–2.55, *p* = 0.28)	0.68 (0.31–1.48, *p* = 0.33)
Age (in years)	1.00 (0.98–1.03, *p* = 0.72)	1.00 (0.98–1.02, *p* = 0.99)	1.03 (1.00–1.05, ***p* = 0.04**)	1.00 (0.97–1.03, *p* = 0.85)
Legal status (rejected asylum seekers)	1.08 (0.57–2.03, *p* = 0.82)	0.72 (0.37–1.39, *p* = 0.33)	1.08 (0.56–2.07, *p* = 0.83)	0.72 (0.31–1.71, *p* = 0.46)
Education level (low education level)	0.66 (0.36–1.18, *p* = 0.16)	2.07 (1.11–3.87, ***p* = 0.02**)	0.67 (0.36–1.23, *p* = 0.19)	0.59 (0.28–1.25, *p* = 0.17)
Health literacy (low health literacy)	0.89 (0.49–1.59, *p* = 0.69)	0.67 (0.37–1.21, *p* = 0.18)	1.60 (0.87–2.92, *p* = 0.13)	0.68 (0.32–1.47, *p* = 0.33)
Place of living (community centers)	0.71 (0.40–1.26, *p* = 0.24)	0.93 (0.52–1.66, *p* = 0.80)	1.18 (0.65–2.17, *p* = 0.58)	0.28 (0.13–0.61, ***p* = 0.001**)
French language proficiency (low level)	1.02 (0.55–1.87, *p* = 0.96)	0.71 (0.38–1.32, *p* = 0.28)	1.44 (0.77–2.67, *p* = 0.25)	0.15 (0.04–0.51, ***p* = 0.002**)
Social worker (presence of)	0.57 (0.31–1.06, *p* = 0.08)	0.56 (0.30–1.04, *p* = 0.07)	1.85 (1.00–3.45, *p* = 0.05)	0.79 (0.36–1.74, *p* = 0.56)
At-risk (at least one comorbidity)	1.59 (0.73–3.45, *p* = 0.24)	2.29 (1.06–4.97, ***p* = 0.04**)	1.73 (0.79–3.80, *p* = 0.17)	1.54 (0.61–3.94, *p* = 0.36)

^a^ A *p*-value < 0.05 is considered statistically significant (in bold in the table).

^b^ Not living home for days at a time.

#### Global Worries and Worries About Access to Care

Our results showed that about 60% of participants were globally worried about the COVID-19 pandemic, and 50% worried about access to medical care. In univariate analyses, ASs without a residence permit were less worried about the COVID-19 pandemic than ASs with an established legal status. (OR 0.5, *p* = 0.044, 95% CI [0.26–0.98]) (Table 3). However, after adjustment, the association between worries about the pandemic and legal status was not statistically significant anymore (aOR 0.48, *p* = 0.072, 95% CI [0.22–1.067]).

**TABLE 3:** Non-adjusted Odd Ratio of participants’ characteristics and COVID-19 pandemic global worry, worry about access to care, associated sleep disorders and fear for one’s life (with 95% CI and *p*-value)^a^ Switzerland, October 2020.

	Global worry	Worry about access to care	Sleep trouble	Fear for life
Gender (female)	1.14 (0.61–2.11, *p* = 0.69)	1.62 (0.89–2.97, *p* = 0.11)	0.69 (0.37–1.27, *p* = 0.23)	1.29 (0.68–2.43, *p* = 0.43)
Age (in years)	0.98 (0.96–1.00, *p* = 0.08)	0.99 (0.96–1.01, *p* = 0.27)	1.01 (0.99–1.03, *p* = 0.39)	1.02 (0.99–1.04, *p* = 0.15)
Legal status (rejected asylum seekers)	0.50 (0.26–0.98, **p = 0.04**)	0.80 (0.42–1.51, *p* = 0.49)	1.35 (0.71–2.57, *p* = 0.37)	1.29 (0.66–2.55, *p* = 0.46)
Education level (low education level)	1.40 (0.74–2.66, *p* = 0.30)	1.37 (0.74–2.54, *p* = 0.32)	0.89 (0.48–1.65, *p* = 0.71)	2.46 (1.30–4.67, ***p* = 0.006**)
Health literacy (low health literacy)	0.85 (0.46–1.58, *p* = 0.61)	1.09 (0.60–1.97, *p* = 0.78)	1.65 (0.90–3.00, *p* = 0.11)	2.09 (1.11–3.94, ***p* = 0.02**)
Place of living (community centers)	0.97 (0.53–1.79, *p* = 0.93)	0.75 (0.41–1.34, *p* = 0.33)	2.04 (1.10–3.79, ***p* = 0.025**)	1.40 (0.73–2.65, *p* = 0.31)
French language proficiency (low level)	0.82 (0.44–1.55, *p* = 0.54)	0.67 (0.36–1.25, *p* = 0.21)	1.36 (0.73–2.53, *p* = 0.33)	1.13 (0.59–2.17, *p* = 0.72)
Social worker (presence of)	0.65 (0.35–1.21, *p* = 0.17)	0.76 (0.41–1.39, *p* = 0.37)	0.75 (0.40–1.40, *p* = 0.36)	1.22 (0.64–2.34, *p* = 0.55)
At-risk (at least one comorbidity)	1.04 (0.46–2.34, *p* = 0.92)	2.28 (0.96–5.39, *p* = 0.06)	1.67 (0.75–3.72, *p* = 0.21)	1.21 (0.51–2.85, *p* = 0.66)

^a^ A *p*-value < 0.05 is considered statistically significant (in bold in the table).

ASs with at least one clinical risk factor for COVID-19 complications were more worried about access to medical care than those without clinical risk factors (aOR 3.33, *p* = 0.017, 95% CI [1.23–8.95]).

#### Sleep Disorders and Fear of Dying

**FIGURE 1:**
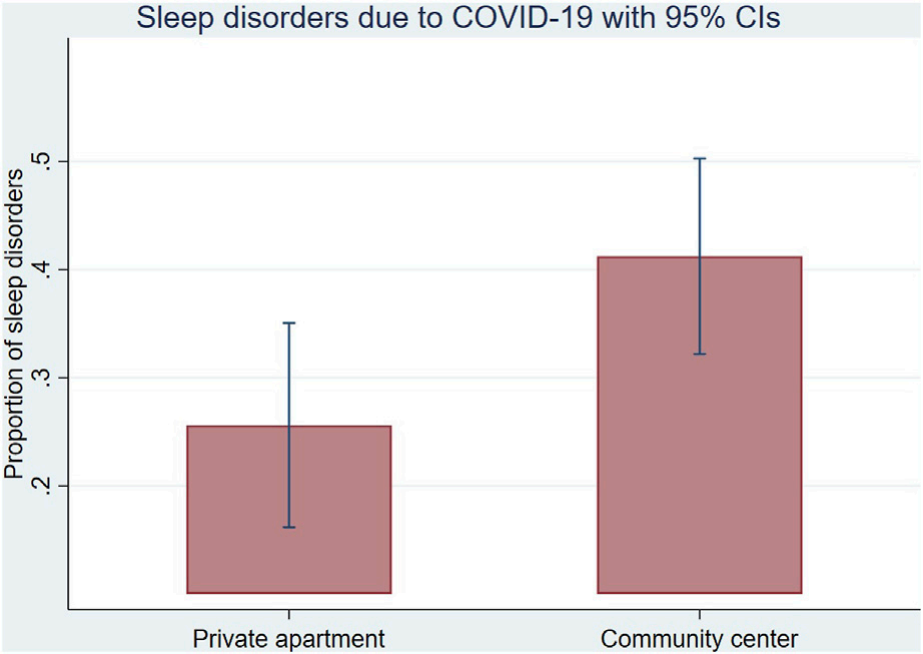
The proportion of sleep disorders due to the COVID-19 pandemic by place of living (with 95% CI) Switzerland, October 2020.

About 35% of participants reported varying degrees of pandemic-related sleep disorders, and 33% agreed or strongly agreed with the statement “I am afraid of losing my life because of the new coronavirus.” The multivariable analyses indicated that participants living in community centers had statistically more sleep disturbances due to the pandemic than participants living in single apartments (aOR 2.21, *p* = 0.023, 95% CI [1.12–4.39]) (Figure 1). Furthermore, in the unadjusted analyses, participants with lower health literacy and lower education were statistically more afraid for their life compared to participants with higher health literacy (OR 2.09, *p* = 0.023, 95% CI [1.11–3.94]) and higher education, respectively (OR 2.46, *p* = 0.006, 95% CI [1.30–4.67]). After adjusting for age, gender, and being at risk of medical complications, participants with lower education remained statistically more afraid of dying of COVID-19 than those with higher education (aOR 2.31, *p* = 0.017, 95% CI [1.16–4.58]). In the subgroup analysis, the presence of a social worker was associated with fewer sleep troubles for males (OR 0.35, *p* = 0.01, 95% CI [0.16–0.80]) but not for females (OR 2.39, *p* = 0.12, 95% CI [0.81–7.06]) (Supplementary Table S2).

### Qualitative Results (Interviews)

Worries among ASs could be grouped into two broad categories. First, shared spaces in residence centers were a significant source of worry for most residents, due to the proximity it imposed on people. Second, as the pandemic put on hold many social activities, it postponed the prospect of obtaining a permit to an uncertain future, generating feelings of isolation and worries about the future, which were heightened among interviewees.

### Shared Spaces

#### Negotiating Risk in Common Spaces

Common areas (i.e., kitchen and bathrooms) were considered a source of worry for many participants. Indeed, except for one, all buildings have shared kitchens and bathrooms. Therefore, due to their living conditions, they could neither follow quarantine measures (no private kitchen or bathroom) nor respect social distances.

Residents developed strategies to deal with their worries, such as avoiding these common spaces. For example, a mother of two children, one with immunodeficiency, was very worried about access to care during the COVID-19 pandemic. According to this participant, her daughter would not have been able to receive her medical treatment if she had contracted the virus. Overseeing her daughter’s health increased her anxiety because the consequence of being contaminated would be double, for herself and her child, whose health was fragile. The participant explained, for instance, that she never took the lift because she was afraid of being in that small area with people who could potentially transmit the virus: “[*About feeling safe when using other spaces than the bedroom*] *Really, I don’t go (laughs) I go only to stairs…*” (Int_2). Even if the regular cleaning reassured her, she avoided common areas as much as possible.

Strategies adopted to limit risks also involved staying outside or cooking early in the morning. Thus, residents changed their daily routines to avoid contact with others, increasing stress and social isolation: “*We have no contact, we do not…go out*” (Int_02). Isolation was reassuring for some of them regarding the risk of contamination but stressful because of the confinement in tiny rooms. In addition, the social and health professionals confirmed that because of the small spaces and the stigmatization infected people experienced, quarantines increased their worries.

#### Others’ Behavior as a Source of Worries

Shared spaces were not only a source of worry because of the increased risk of contamination but also because of their social implications. By sharing personal space, residents were confronted with how others were or were not protecting themselves. Indeed, having to show how well they were complying with the protective measures constantly exposed them to the moral judgment of others. For example, one father judged the others’ behaviors because he was very worried about his family, especially his pregnant wife. He wanted to protect them from the virus that he thought was circulating a lot in the center because of the irresponsibility of the other residents: “*And if besides the fact that they offer you everything* [*talking about the protective equipment provided by the center*]*, you decide not to do it, it’s your fault*” (Int_01). Thus, others’ behaviour led to judgements between residents and increased fear and tensions for some.

Despite worrying about the lack of compliance with protective measures, some residents expressed their understanding regarding the challenge of following those measures in such a setting: “[*About the feeling that people are not respectful of the rules*] *I mean, to some extent, it’s not possible, so*” (Int_11)*.* This participant was not worried about the virus because he did not feel medically at risk of COVID-19 complications and found these measures irrelevant and uncomfortable. However, he still respected them because of the injunction of solidarity to protect others. In addition, some reported that, sometimes, more than ten residents living on the same floor could be in the same kitchen cooking. In those cases, maintaining social distancing and wearing a mask could be difficult, if not impossible, to enforce. These two examples illustrate the complexity of risk reduction in crowded spaces and the differences in understanding risk and protective measures.

### Isolated From the Social and Workspace

#### Sudden Cessation of Educational and Leisure Activities and Job-Seeking

Most social activities of ASs were temporarily suspended during lockdowns. Residents could not search for work, and some lost their jobs. They could no longer practice physical activities or take French classes at the center: “*When COVID came, I was in my room, with no job, and no French classes. For almost ten months like that*” (Int_08). The COVID-19 period was complicated for this man because he felt disconnected from the world. He feared he would no longer have a job and would be unable to progress in French. These occupations usually allow residents to occupy themselves and create a weekly rhythm. Stopping these activities meant not being able to find a job and become socially integrated:

*It is co*mplicated, even if I make 200-300 job offers, and then I am told, *“*it's interesting, but there aren't any; we're sorry*."* If there were no virus, maybe I would have found a job. But now it's complicated. (...) I really want to work because I have been locked up for a year and a half (Int_07).

It is not a priority for me because I came here, I thought I would start a new life; there are many things for me to do, to build a life, (...) there are other problems more important than COVID. (...) Yes, I want to study to build my life (Int_06).

As the last interviewee expressed, avoiding COVID-19 was not a priority for most ASs because they had other priorities, like constructing a new life in Switzerland and getting a permit. This participant had a good social network because he was taking dance classes, but these were utterly closed, so he had to find alternative activities during the pandemic (e.g., watching series to learn French).

Residents have lost their social contact inside and outside the center. This isolation was excruciating for some: “*In terms of mental health, I see the place as a prison*.” (Int_07). Living with his two brothers in one bedroom, he felt trapped and isolated.

These experiences illustrate the challenges of social isolation during the COVID-19 pandemic and its impact on social integration. This situation also generated uncertainty about ASs’ future in Switzerland and in the center, as they had little or no control over the situation.

#### Being Separated From the Family

The pandemic also increased the isolation of the residents from their families:

I was really isolated, and in my exile, I was cut off from my family, from my son and my wife, we had telephone contact, and then the telephone I had was not a very good telephone; the connection was a problem (Int_10).

Here the participant, who left his wife and child behind in his home country, explained that regular phone contact was impossible due to an unusual problem with the internet connection and described his experience of being away from his family as an exile. If the separation from the family is generally perceived as painful for most ASs, it was exacerbated by the pandemic as they were cut off from other social contacts. Although their permit does not allow them to visit their families in their home country, the need to be with them was much more vital given the complex health situation. In particular, they worried about the impact of the pandemic on their relatives and their health due to the fragility of health systems in their home country. The lack of contact and distance with their families were therefore experienced as an essential missing resource, which aggravated their worries during the pandemic.

## Discussion

Our mixed-method study highlighted that the experiences and worries of AS during the COVID-19 pandemic were influenced by social determinants of health, such as gender, living conditions and education. We could observe two main forms of worry. For some participants, the worries focused on virus contamination and disease. For those, the worries were generated mainly by shared spaces, the inappropriateness of measures in community centres, and their inability to protect themselves sufficiently due to living conditions. In contrast, others worried about the consequences of public health measures and what it meant for their wellbeing and life perspectives. Moreover, we found that a significant minority of participants reported no specific worry about the pandemic and associated protective measures. Based on our interviews, we could hypothesize that their worries were more oriented towards other priorities, for instance, access to language courses, getting a job and maintaining social contacts. These divergent positions were also generated by protective measures and how people perceive the risks and decide to react to them.

Our findings confirm previous studies highlighting the clinical and social impact of the COVID-19 pandemic on migrant populations. First, four cross-sectional surveys highlighted the mental health burden of the COVID-19 pandemic on refugees [19, 20, 27] and migrant workers [28]. One found, for example, that 78.7% of participants suffered a decrease in their wellbeing since the beginning of the pandemic [19]. Then, a mixed-method study showed a high prevalence of exposure to COVID-19, poor mental health, and frequent avoidance of healthcare among undocumented migrants [9]. Similarly, a qualitative study described the social and economic burden of the COVID-19 pandemic experienced by migrant populations [21]. Regarding the perception of migrant populations towards public health measures, such as lockdowns, our study confirms the ambivalent feelings also observed in shelters in France. For some people, lockdown was perceived as positive because of the security it provided against the COVID-19 infection. For others, it was incompatible with their living conditions and affected them negatively [18].

Another significant result of our study shows that AS living in centers had significantly more sleep disorders due to the COVID-19 pandemic than those living in single apartments. These results suggest a higher burden of living conditions on the pandemic experience. If space was perceived as problematic during the pandemic for the general population because of social isolation, it was even more challenging for AS in community centers where space was reduced and shared, creating an increased risk for contamination. These findings confirmed the results of a large online international survey among AS and refugees, where asylum centre participants reported a higher sleep deterioration than those living in a single apartment [29].

Moreover, worries were not only generated by shared spaces but also by social isolation and loss of social resources. Most social activities had to be stopped, restraining residents in their socialization. This suspended time, described as a prison by some participants, completely disconnected them from the society where they were trying to construct a new life. For AS, the feeling that their lives were “on hold” had negative consequences for their emotional and physical health, likely compounded by the restrictions associated with the pandemic [30].

Living in a center became a factor of clinical (increased risk of contamination), psychological (more anxiety), and social (isolation) vulnerability. To cope with it, participants developed strategies to avoid common spaces, as also identified in a previous study [18]. Moreover, because of the protective measures, residents were disconnected from and had to reorganize their social lives, resulting in increased social isolation for some.

Then, our study highlighted the higher economic impact of the COVID-19 pandemic and associated measures on AS with high French proficiencies and those living in single apartments. These unintuitive results mainly reflected the difficulties for AS with low language competencies and those living in community centers to access the work market before the COVID-19 pandemic.

Eventually, the subgroup analysis described gender-related differences in the pandemic experience. Specifically, social workers appeared to be a protective factor for male participants regarding loneliness, social isolation and sleep trouble, but not for females. Further research is needed to better understand these results.

Our study has some limitations. First, the cross-sectional survey did not allow the assessment of changes over time, which precludes drawing temporal associations. Second, our survey may be subject to desirability bias because residents were self-selected for participation, although anonymity should limit this risk. Third, our study had potential selection bias. Indeed, participants may have a higher level of education or social integration than the overall population of AS and refugees in the Canton of Vaud. However, thanks to the collaboration with NGOs and cantonal asylum authorities and the translation of the questionnaires into ten languages, we hope to have limited this bias. Fourth, due to the rapid turnover of residents in community centers, participants who answered the survey differed from those who underwent qualitative interviews. We considered their situations and conditions similar because both were AS living in the same region of Switzerland. Fifth, we restricted the interviews to one center because of the challenges in accessing fieldwork during the pandemic. Nevertheless, a saturation level was reached for this center. Finally, although ethnographic field observations were initially planned, we restricted them during the interview visits due to the pandemic.

In conclusion, our study highlights the importance of proposing public health measures adapted to the needs of asylum seekers and their living conditions at the outset of a crisis such as the COVID-19 pandemic. Such measures could include: avoiding high-density facilities and encouraging the transfer of asylum seekers from community centers to private facilities, ensuring the applicability of measures such as quarantine and isolation in the different living places of asylum seekers, adapting the communication of health recommendations for asylum seekers, managing mental health with preventive actions and adapting (instead of cancelling) social activities to the pandemic protective measures. Policymakers should also consider addressing adverse social and structural determinants of the health of asylum seekers through fair asylum policies, good living conditions, and full access to care. These results have served as a basis for developing recommendations for local authorities and professionals. Worries about overcrowding and social isolation were recognized by the professionals interviewed, who are also willing to make changes within their facilities in line with the proposed recommendations and with the support of the authorities.
