# Supplementary material for: Facing the COVID-19 Pandemic: A Mixed-Method Analysis of Asylum Seekers’ Experiences and Worries in the Canton of Vaud, Switzerland
Source: Int J Public Health. 2023 Sep 27;68:1606229. doi: 10.3389/ijph.2023.1606229 (PMC10564980; doi:10.3389/ijph.2023.1606229)
Supplement: Supplementary file 1 [file DataSheet2.PDF]

Additional file 2

Interview participants' characteristics

Interview number	Age	Gender	Interview language	Place of living
01	31 years old	Man	Spanish	Community center
02	37 years old and unknown	2 women	English	Community center
03	17 years old	Man	French	Community center
04	42 years old	Man	Dari	Community center
05	36 years old	Woman	Turkish	Community center
06	37 years old	Man	French	Community center
07	28 years old	Man	Albanian	Community center
08	34 years old	Man	French	Community center
09	46 years old	Man	French	Community center
10	46 years old	Man	French	Community center
11	36 years old	Man	English	Community center
12	About 40 years old	Man & woman	Georgian	Community center
13	Unknown	Woman	French	Community center
