# Supplementary material for: Facing the COVID-19 Pandemic: A Mixed-Method Analysis of Asylum Seekers’ Experiences and Worries in the Canton of Vaud, Switzerland
Source: Int J Public Health. 2023 Sep 27;68:1606229. doi: 10.3389/ijph.2023.1606229 (PMC10564980; doi:10.3389/ijph.2023.1606229)
Supplement: Supplementary file 2 [file Table1.docx]

**Additional Table 1 Non-adjusted Odd Ratio of participants’ characteristics and COVID-19 pandemic impact on social isolation, loneliness, anxiety and economic losses (with 95% CI and p-value)^[[1]](#footnote-1)^, by gender**

| **Male** | Social Isolation^[[2]](#footnote-2)^ | Loneliness | Anxiety | Economic losses |
| --- | --- | --- | --- | --- |
| **Age (in years)** | 0.99 (0.96-1.02, p=0.50) | 1.00 (0.97-1.03, p=0.98) | 1.02 (0.99-1.06, p=0.23) | 1.00 (0.96-1.04, p=0.95) |
| **Legal status (Rejected asylum seekers)** | 0.76 (0.35-1.66, p=0.50) | 0.77 (0.34-1.72, p=0.52) | 0.98 (0.43-2.26, p=0.97) | 0.58 (0.21-1.60, p=0.29) |
| **Education level (Low education level)** | 0.99 (0.47-2.10, p=0.98) | 2.18 (0.97-4.88, p=0.06) | 1.51 (0.66-3.43, p=0.33) | 0.89 (0.35-2.23, p=0.80) |
| **Health literacy (Low health literacy)** | 0.62 (0.29-1.31, p=0.21) | 0.58 (0.27-1.25, p=0.16) | 1.41 (0.64-3.11, p=0.39) | 0.35 (0.13-0.97, **p=0.04**) |
| **Place of living (Community centers)** | 0.54 (0.26-1.13, p=0.10) | 0.65 (0.30-1.37, p=0.26) | 0.73 (0.33-1.60, p=0.43) | 0.11 (0.03-0.34, **p<0.001**) |
| **French language proficiency (Low level)** | 0.96 (0.44-2.10, p=0.91) | 0.63 (0.28-1.45, p=0.28) | 1.34 (0.59-3.10, p=0.49) | 0.15 (0.03-0.70, **p=0.015**) |
| **Social worker (presence of)** | 0.41 (0.19-0.88, **p=0.02**) | 0.34 (0.15-0.77, **p=0.01**) | 1.13 (0.50-2.57, p=0.77) | 0.56 (0.21-1.46, p=0.24) |
| **At risk (at least one comorbidity)** | 1.46 (0.52-4.10, p=0.23) | 1.89 (0.68-5.33, p=0.23) | 1.06 (0.34-3.29, p=0.92) | 1.36 (0.40-4.64, p=0.62) |

| **Female** | Social Isolation^[[3]](#footnote-3)^ | Loneliness | Anxiety | Economic losses |
| --- | --- | --- | --- | --- |
| **Age (in years)** | 1.02 (0.99-1.06, p=0.20) | 1.00 (0.97-1.04, p=1.00) | 1.03 (0.99-1.07, p=0.10) | 1.01 (0.96-1.05, p=0.71) |
| **Legal status (Rejected asylum seekers)** | 1.59 (0.49-5.13, p=0.44) | 0.56 (0.17-1.97, p=0.37) | 1.67 (0.53-5.23, p=0.38) | 1.02 (0.19-5.38, p=0.98) |
| **Education level (Low education level)** | 0.32 (0.19-0.86, **p=0.02**) | 1.72 (0.63-4.67, p=0.29) | 0.21 (0.08-0.57, **p=0.002**) | 0.29 (0.08-1.08, p=0.07) |
| **Health literacy (Low health literacy)** | 1.60 (0.61-4.21, p=0.34) | 0.94 (0.36-2.42, p=0.90) | 1.88 (0.73-4.85, p=0.19) | 1.97 (0.54-7.13, p=0.30) |
| **Place of living (Community centers)** | 1.08 (0.41-2.82, p=0.88) | 1.43 (0.55-3.68, p=0.46) | 2.52 (0.95-6.74, p=0.07) | 1.27 (0.34-4.74, p=0.72) |
| **French language proficiency (Low level)** | 1.30 (0.49-3.46, p=0.60) | 0.77 (0.29-2.05, p=0.61) | 1.59 (0.61-4.14, p=0.34) | 0.15 (0.02-1.23, p=0.08) |
| **Social worker (presence of)** | 1.00 (0.36-2.80, p=1.00) | 1.19 (0.44-3.18, p=0.74) | 4.75 (1.70-13.31, **p=0.003**) | 1.40 (0.36-5.48, p=0.63) |
| **At-risk (at least one comorbidity)** | 2.17 (0.64-7.28, p=0.21) | 2.56 (0.76-8.54, p=0.13) | 3.50 (1.02-12.00, **p=0.046**) | 2.21 (0.50-9.78, p=0.30) |

1. A p-value < 0.05 is considered statistically significant (in bold in the table) [↑](#footnote-ref-1)
2. Not living home for days at a time [↑](#footnote-ref-2)
3. Not living home for days at a time [↑](#footnote-ref-3)
