# Supplementary material for: Facing the COVID-19 Pandemic: A Mixed-Method Analysis of Asylum Seekers’ Experiences and Worries in the Canton of Vaud, Switzerland
Source: Int J Public Health. 2023 Sep 27;68:1606229. doi: 10.3389/ijph.2023.1606229 (PMC10564980; doi:10.3389/ijph.2023.1606229)
Supplement: Supplementary file 3 [file DataSheet3.PDF]

Additional file 3

Interview guide: Experience of the pandemic during the different waves and the difficulties encountered

**General topics (all populations)**
Perception of risk (of exposure and transmission): *How do you feel about the epidemic and the associated risks?*<br>• Risk of exposure/transmission<br>• Risk to self/others<br>• Monitoring developments (sources of information, reliability)
Experience with protective measures: *What do you think of the recommended protective measures and how do you feel about them?*<br>• Measures perceived as essential<br>• Impact of measures on daily life<br>• Adoption of/adherence to measures and easiness/difficulty in applying them<br>• Experience of these measures<br>• Change in practice/application of measures over time
Experience of the disease (if applicable): *What happened when you contracted the disease and how did you cope?*<br>• Experience of the disease<br>• Protective measures for yourself/others and how easy/difficult they were to put in place<br>• Impact on daily life (organisation of activities, social contacts)<br>• Stigma/guilt about the disease, reactions of family and friends<br>• Changes between before and after the illness (e.g. in the adoption of protective measures)
Impact and changes (positive, negative, new difficulties/problems, new habits, etc.): *What changes and consequences do you see the pandemic having for you and/or those close to you?*<br>• Impact of the pandemic (in general, points, etc.)<br>• Consequences on health (in general, physical, psychological)<br>• Changes (appearance of new difficulties/problems, new habits)
Impact on social relationships: *How has the pandemic affected your social relationships?*<br>• Significant people in your daily life<br>• Impact of the pandemic (difficulties/facilities, changes in friendships/family relationships - maintenance, closeness, distance)

Future perspectives and expectations, general feelings and possibilities for development: *What is your overall impression of the pandemic so far, and how do you see it developing?*<br>- Evolution of the pandemic (coming months)<br>- Satisfaction with the measures taken for the general population<br>- Expectations for the coming months (health & economic crisis management, role of health institutions/state/public policy, actions/behaviour of the general population, new measures such as vaccines/rapid testing)

**Specific topics (asylum seekers)**
Experience of the pandemic in the community center: *How did you experience/are you experiencing the pandemic/confinement in this center?*<br>• Perception of the living space (people with whom the spaces are shared, suitability of the space for the containment/pandemic)<br>• Implementation of protection measures (management of different areas, changes in practices over time, appropriateness of measures taken, experience)
Impact and changes (positive, negative, new difficulties/problems, new habits, etc.): *What changes and consequences do you see the pandemic having for you and/or those close to you?*<br>• Impact on the migration/asylum process (living environment, protective measures)
