# Supplementary material for: Facing the COVID-19 Pandemic: A Mixed-Method Analysis of Asylum Seekers’ Experiences and Worries in the Canton of Vaud, Switzerland
Source: Int J Public Health. 2023 Sep 27;68:1606229. doi: 10.3389/ijph.2023.1606229 (PMC10564980; doi:10.3389/ijph.2023.1606229)
Supplement: Supplementary file 4 [file Table2.docx]

**Additional Table 2 Non-adjusted Odd Ratio of participants’ characteristics and COVID-19 pandemic global worry, worry about access to care, associated sleep disorders and fear for one’s life (with 95% CI and p-value)^[[1]](#footnote-1)^, by gender**

| **Male** | Global worry | Worry about access to care | Sleep trouble | Fear for life |
| --- | --- | --- | --- | --- |
| **Age (in years)** | 0.99 (0.96-1.02, p=0.61) | 0.98 (0.94-1.01, p=0.17) | 1.01 (0.98-1.04, p=0.63) | 1.02 (0.99-1.06, p=0.25) |
| **Legal status (Rejected asylum seekers)** | 0.57 (0.25-1.29, p=0.18) | 1.07 (0.49-2.32, p=0.87) | 0.85 (0.38-1.89, p=0.69) | 1.80 (0.75-4.28, p=0.19) |
| **Education level (Low education level)** | 1.17 (0.53-2.57, p=0.69) | 1.65 (0.76-3.59, p=0.21) | 1.07 (0.49-2.30, p=0.87) | 2.18 (0.87-5.43, p=0.10) |
| **Health literacy (Low health literacy)** | 0.78 (0.36-1.71, p=0.54) | 0.81 (0.38-1.74, p=0.59) | 1.48 (0.70-3.16, p=0.31) | 1.96 (0.84-4.55, p=0.12) |
| **Place of living (Community centers)** | 0.69 (0.32-1.50, p=0.35) | 0.41 (0.19-0.88, **p=0.02**) | 1.43 (0.67-3.05, p=0.36) | 1.87 (0.78-4.45, p=0.16) |
| **French language proficiency (Low level)** | 1.28 (0.56-2.91, p=0.55) | 0.72 (0.32-1.63, p=0.43) | 1.40 (0.63-3.10, p=0.41) | 1.29 (0.54-3.10, p=0.57) |
| **Social worker (presence of)** | 0.55 (0.25-1.21, p=0.14) | 0.51 (0.23-1.12, p=0.09) | 0.35 (0.16-0.80, **p=0.01**) | 1.44 (0.61-3.42, p=0.41) |
| **At risk (at least one comorbidity)** | 0.86 (0.30-2.42, p=0.77) | 2.35 (0.79-7.01, p=0.13) | 2.09 (0.74-5.89, p=0.16) | 0.57 (0.15-2.25, p=0.41) |

| **Female** | Global worry | Worry about access to care | Sleep trouble | Fear for life |
| --- | --- | --- | --- | --- |
| **Age (in years)** | 0.96 (0.93-1.00, p=0.052) | 0.99 (0.96-1.03, p=0.76) | 1.01 (0.98-1.05, p=0.44) | 1.02 (0.98-1.05, p=0.36) |
| **Legal status (Rejected asylum seekers)** | 0.36 (0.10-1.29, p=0.12) | 0.52 (0.15-1.77, p=0.29) | 3.14 (0.94-10.52, p=0.06) | 0.71 (0.20-2.51, p=0.59) |
| **Education level (Low education level)** | 1.53 (0.56-4.17, p=0.41) | 1.44 (0.54-3.82, p=0.47) | 0.36 (0.13-1.03, p=0.06) | 1.4 (0.51-3.87, p=0.52) |
| **Health literacy (Low health literacy)** | 1.10 (0.39-3.13, p=0.86) | 2.23 (0.79-6.24, p=0.13) | 2.26 (0.81-6.37, p=0.12) | 2.59 (0.95-7.01, p=0.06) |
| **Place of living (Community centers)** | 1.55 (0.58-4.16, p=0.39) | 1.63 (0.62-4.28, p=0.33) | 4.18 (1.25-13.98, **p=0.02**) | 0.89 (0.33-2.37, p=0.82) |
| **French language proficiency (Low level)** | 0.38 (0.14-1.09, p=0.07) | 0.53 (0.20-1.42, p=0.21) | 1.34 (0.48-3.76, p=0.58) | 1.04 (0.38-2.83, p=0.94) |
| **Social worker (presence of)** | 0.92 (0.31-2.75, p=0.89) | 1.75 (0.59-5.15, p=0.31) | 2.39 (0.81-7.06, p=0.12) | 1.24 (0.44-3.56, p=0.68) |
| **At risk (at least one comorbidity)** | 1.22 (0.32-4.65, p=0.77) | 1.91 (0.45-8.10, p=0.38) | 0.91 (0.22-3.80, p=0.90) | 3.27 (0.89-12.03, p=0.08) |

1. A p-value < 0.05 is considered statistically significant (in bold in the table) [↑](#footnote-ref-1)
