# Supplementary material for: Facing the COVID-19 Pandemic: A Mixed-Method Analysis of Asylum Seekers’ Experiences and Worries in the Canton of Vaud, Switzerland
Source: Int J Public Health. 2023 Sep 27;68:1606229. doi: 10.3389/ijph.2023.1606229 (PMC10564980; doi:10.3389/ijph.2023.1606229)
Supplement: Supplementary file 5 [file DataSheet1.PDF]

Anglais

unisanté

Centre universitaire de médecine générale et santé publique · Lausanne

Coronavirus Response in the Vaud Canton

**My preferred language is:**<br>**Ma langue préférée est:**<br>**Moj omiljeni jezik je:**<br>**لغتي المفضلة هي**<br>**زبان مورد علاقه من است**<br>**ዝመርጾ ቋንቋ**<br>**எனக்கு விருப்பமான மொழி:**<br>**ჩემი საყვარელი ენა არის**<br>**Gjuha ime e preferuar është:**<br>**Luuqada aan ugu jeclahay waa**	(•) English<br>( ) Français<br>( ) SR/HR/BIH<br>( ) اللغة العربية<br>( ) فارسی<br>( ) ትግርኛ<br>( ) தமிழ், இலங்கை<br>( ) ქართული<br>( ) gjuha shqipe<br>( ) Soomaali

Coronavirus Response in the Vaud Canton

We would like to know how you feel about the new coronavirus.

We would like to know about the impact of the new coronavirus on your life. We would also like to know how you feel about the recommendations to contain it.

The answers you provide will be used to improve the Canton responses and to help research in this area.

It is up to you whether or not you want to take part. Taking part is anonymous. This means we do not know your name. We will not ask for personal information that can identify you.

We will not contact you again after this survey.

Thank you for your time.

1	**What is your age? (in years)**	__________
2	**What is your sex?**	( ) Male<br>( ) Female

- Other

3 How well do you speak French?

- Very well
- Well
- Not well
- Not at all
- I don't know

4 What type of housing do you live in?

- Private apartment or house
- Room in asylum seeker center with USMi consultation
- Room in asylum seeker center without USMi consultation
- I don't know

5 Do you have:

- N Permit
- F Permit
- B Permit
- S Permit
- Emergency aid (white paper)
- Swiss citizenship or C Permit
- I don't know

6 Do you get help from a social worker, community advocate or someone else?

- Yes
- No
- I don't know

7 What is your highest level of education?

- Compulsory education or less
- Apprenticeship
- High school
- Professional school or university
- I don't know

8 How confident are you filling out forms about your health by yourself?

- Always
- Often
- Sometimes
- Occasionally
- Never

9 Have you been tested for the new coronavirus?

- Yes, I tested positive
- Yes, I tested negative

- Yes, I am awaiting the result
- No
- I don't know

10 Are you part of a group at risk for the new coronavirus?
This means that you have hypertension, diabetes, heart problems, lung problems (ex: asthma or chronic bronchitis) or have a weaker immune system (immunosuppressed).

- Yes
- No
- I don't know

11 Do you know what to do if you have coronavirus symptoms?

I really don't know what to do — I know exactly what to do

0........1........2........3........4........5........6........7........8........9........10

12 How have you stayed up to date with recommendations for the new coronavirus?
(Check all responses that apply)

- I watched the television or listened to the radio
- I looked at government websites
- I listened to government announcements
- I received information from community interpreters where I live
- I read the news online or in a newspaper
- I looked at social media websites (Facebook, Instagram, WhatsApp, Twitter, etc)
- I got information from my employer
- I got information from my family and friends
- I called the government telephone hotline
- I spoke to healthcare professionals
- I looked at hospital or clinic websites
- I have not stayed up to date about the coronavirus
- I don't know

13 Which of the following things are currently recommended by the government to slow the spread of the new coronavirus?
(Check all responses that apply)

- Stay outside as long as possible each day
- If you have a fever or cough, stay home for 10 days and 48 hours without symptoms
- Stop using public transports
- Stay 1.5 meters away from others
- Spontaneous gatherings of up to 30 people are allowed
- Work at home as much as you can
- Shops selling clothes are now opened

- [ ] bars and pubs are opened to all if clients are standing up or outside on a terrace
- [ ] I don't know

14 Do you think that the following statements are true:
(Check all responses that apply)

- [ ] The new coronavirus was created intentionally in a laboratory
- [ ] The new coronavirus occurred naturally due to mixing of human and animal viruses
- [ ] The new coronavirus was created as a weapon, probably by China or the United States
- [ ] The new coronavirus has disproportionately affected poorer people
- [ ] The effects of the coronavirus have been intentionally exaggerated so that governments can better control their populations
- [ ] The new coronavirus was created to scare asylum seekers and make them leave
- [ ] I don't know

15 How much have you followed the government recommendations?

not at all — all the time

0........1........2........3........4........5........6........7........8........9........10

16 What has stopped you from following the government recommendations?
(Check all responses that apply)

- [ ] I don't think the recommendations are useful
- [ ] I don't think the virus is dangerous for me or my family
- [ ] I don't have a choice (ex: I must keep working)
- [ ] I don't have the means (ex: I live in shared space)
- [ ] My home is too small to stay inside all the time
- [ ] I have to care for my family (children or older people)
- [ ] I had to leave the house for food and other essentials
- [ ] I worry about offending others (ex: not shaking hands when offered)
- [ ] I worry about my health (ex: I need to see a doctor)
- [ ] I don't remember the recommendations
- [ ] I don't understand the recommendations
- [ ] I don't know

17 Do you feel the government recommendations have been:

not at all — about — much too

enough right restrictive

0........1........2........3........4........5........6........7........8........9........10

18 What impact have the new coronavirus restrictions had on your life?
(check all responses that apply)

- ☐ I have lost money
- ☐ I have lost my job
- ☐ I had to close my business
- ☐ I have kept my job, but lost money because of missed hours of work
- ☐ I have been more lonely
- ☐ I have been more anxious
- ☐ I have lost a loved one to coronavirus
- ☐ I did not leave my home for days at a time
- ☐ Not a big impact on my life
- ☐ I don't know

19 Before the new coronavirus:
(check all responses that apply)

- ☐ I saw a doctor or nurse for my physical health problems
- ☐ I saw a doctor or nurse for my mental health problems
- ☐ I did not see a doctor or nurse
- ☐ I don't know

Since the new coronavirus started, I have seen a doctor or nurse for my physical health problems:
(check all responses that apply)

- ☐ As much as before
- ☐ Less than before
- ☐ At the doctor or nurse office
- ☐ On the phone
- ☐ On video
- ☐ Not at all
- ☐ I don't know

Since the new coronavirus started, I have seen a doctor or nurse for my mental health problems:
(check all responses that apply)

- ☐ As much as before
- ☐ Less than before
- ☐ At the doctor or nurse office
- ☐ On the phone
- ☐ On video
- ☐ Not at all

20 Over the past 3 months, I have been worried about the new coronavirus:

not worried at all — extremely worried

0........1........2........3........4........5........6........7........8........9........10

21 I had trouble falling or staying asleep because I was thinking about the new coronavirus:

- Not at all
- Rare, less than a day or two
- Several days
- More than 7 days
- Nearly every day over the last two weeks
- I don't know

22 I am afraid of losing my life because of the new coronavirus:

- Strongly disagree
- Disagree
- Neither agree nor disagree
- Agree
- Strongly agree
- I don't know

23 I have worried during the past three months about access to medical care if infected by coronavirus:

not worried at all — extremely worried

0........1........2........3........4........5........6........7........8........9........10
